# Heating Power of Low Charges of Electricity

**Published:** 1848-09

**Authors:** 


*Heating Power of Low Charges of Electricity.*—In a lecture delivered at the Royal Institution, on Saturday last, Mr. Faraday demonstrated by a simple experiment that coal-gas might be ignited by a very feeble electric spark. He insulated on a glass stand a globular iron vessel containing the condensed gas. He then applied to it, twice, a glass tube which had been each time rubbed with a hare-skin. On applying the point of the forefinger near to the mouth of the jet from which the gas was escaping, the small spark which escaped was sufficient to ignite the gas.—*Ibid.*

